# Massive Myocardial Infarction in a Full-Term Newborn: A Case Report

**DOI:** 10.1155/2010/658065

**Published:** 2010-06-16

**Authors:** Vlasta Fesslova, Gina Lucci, Jelena Brankovic, Stefania Cordaro, Emilio Caselli, Guido Moro

**Affiliations:** ^1^Center of Fetal Cardiology, Policlinico San Donato IRCCS, 20097 Milan, Italy; ^2^Neonatal Intensive Care Unit, P.O. Macedonio Melloni, Italy; ^3^Pathologic Anatomy Section, P.O. Macedonio Melloni, Milan, Italy

## Abstract

A full-term female newborn with neonatal asphyxia and severe anemia (Hb 2.5 g/dL) with normal heart developed a massive myocardial infarction. No examinations were performed during pregnancy for parental nomadism. The baby had immediate external cardiac massage, ventilatory assistance, and blood transfusion. Cardiomegaly was evident at chest X-ray and marked signs of ischemia-lesion at ECG. Echocardiography showed dilated, hypertrophic, and hypocontractile left ventricle (LV), mitral and tricuspid regurgitation, and moderate pericardial effusion. Rh isoimmunization and infective agents were excluded at laboratory tests. Despite the treatment with inotropes, hydrocortisone, and furosemide, the baby worsened and died at 45 hours of life. Postmortem examination showed diffuse subendocardial infarction of LV and diffuse parenchymal hemorrhages and myocardial hypertrophy, increase of eosinophilia, and polymorphonucleated cells at histology. Our patient suffered apparently from longstanding fetal anemia of unknown etiology that led to perinatal distress, severe hypoxia, and massive myocardial infarction, unresponsive to the therapy.

## 1. Introduction

The occurrence of myocardial infarction in newborns has been associated with cardiac malformations and abnormalities of the coronary arteries or thromboembolism [1]. The syndrome of hypoxemia-related myocardial dysfunction in newborns occurs in about 30% of asphyxiated infants and is usually more relevant in preterm infants, due to immature myocardial contractility and respiratory distress syndrome [2]. Myocardial ischemia in full term infants with normal heart is an extremely rare and serious event.

We report a case of a full term newborn affected by neonatal asphyxia and severe anemia, with normal heart and coronary arteries anatomy, who developed a massive myocardial infarction.

## 2. Case Report

A female baby GDG was born at 38 weeks of gestation by spontaneous delivery. No examinations were performed during pregnancy for parental nomadism. Birth weight was 3470 g and Apgar score 1/3/3. After delivery, the baby needed immediate cardiopulmonary resuscitation with intubation, external cardiac massage, ventilatory assistance (A/C with PIP 20, PEEP +5, TI 350 msec, FR 50/minute, MAP 9.9, FiO_2_ 0.80), and an immediate blood transfusion for severe anemia (Hb 2.5 g/dL). Severe metabolic acidosis was present (pH 6.81), with arterial hypotension (41/19 mmHg). Correction of acidosis and treatment with dopamine, dobutamine, hydrocortisone, and furosemide was started. 

**Figure 1:**
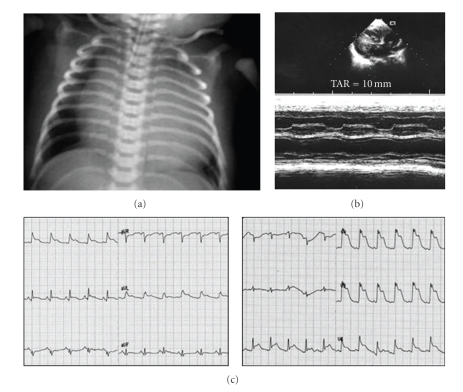
(a) Chest X-ray—marked cardiomegaly, (b) 2-D and M-mode echocardiography showing biventricular hypertrophy with left ventricular hypokinesis (c) ECG—signs of diffuse subendocardial ischemia-lesion.

Chest X-ray showed marked cardiomegaly (Figure 1a).

On ECG there were small Q waves in D2-3 and marked signs of subendocardial ischemia-lesion—with diffused ST-T segment elevation (Figure 1c).

Echocardiography (Figure 1b) showed normal anatomy with the coronary arteries originated from the aorta; dilated and hypocontratile left ventricle (LV), with hypertrophic walls, mitral regurgitation, dilated right ventricle with tricuspid regurgitation and estimated pulmonary pressure of 35 mmHg, left-to-right shunt at atrial and ductal level, and moderate pericardial effusion. 

Serum enzymes, at 10 hours of life, were: CK = 14.895 U/L, CK-MB = 2.472 U/L and LDH = 3771 U/L. Rh isoimmunization was excluded and no specific infective agents were found by laboratory tests. 

Despite of the treatment, the clinical conditions of the baby worsened, with appearance of anuria, hepatic insufficiency, massive haemorrhage of the right cerebral hemisphere, and disseminated intravascular coagulation. The baby died at 45 hours of life.

**Figure 2:**
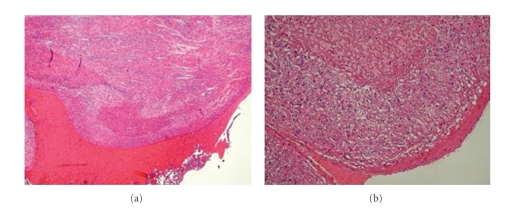
(a) Histology—showing a zone of myocardial infarction, (b) Histology—an increase of eosinophilia.

Post-mortem examination (Figure 2) showed massive subendocardial infarction of the LV, diffused parenchymal hemorrhages in lungs, kidneys, adrenal glands, and brain; with histological appearance of myocardial hypertrophy, increase of eosinophilia and polymorphonucleated cells.

## 3. Discussion

Myocardial ischemia is a rare event in childhood and can occur in the immediate postnatal period, usually due to perinatal asphyxia and respiratory problems. This complication is more frequent in premature infants with severe respiratory distress [2] that present abnormal left ventricular function and abnormal values of CK and CK-MB. Previously, a rare case of myocardial necrosis-infarction was reported in a premature infant with asphyxia, and the infant recovered, using the conventional treatment [3]. In full term infants, the etiology of myocardial dysfunction-ischemia is mostly related to asphyxia. Another case was reported of a full term infant who presented at 12 days of age with myocardial infarction complicated by late mitral regurgitation and heart failure that improved after mitral valve annuloplasty at 6 months of age [4]. Our case suffered apparently from a longstanding anemia of unknown etiology, also due to a lack of routine controls in pregnancy; this fact induced a compensatory mechanism of myocardial hypertrophy that was observed at echocardiography and at post-mortem examination. The entity of anemia has worsened toward the end of pregnancy (as evident from the very low value of haemoglobin) and led to a severe fetal and postnatal distress due to the severe hypoxia. Myocardial-subendocardial necrosis was very diffused and the general state was very severe since the moment of birth, and all the therapeutic measures remained inefficacious. Neither a hypothetical use of ECMO, reported to be employed in another full term infant who presented however later on, at 9 days of age [5], could have saved our infant with a more precocious and severe symptomatology.

## 4. Conclusions

Our case regards a very unusual event of a massive myocardial infarction in a full term neonate that occurred on the basis of a longstanding fetal anemia of unknown etiology leading to the perinatal distress with severe hypoxia and multiorgan failure not improvable by the treatment.
